# Coffee, the Gut Microbiome, and Host Metabolism: Gastrointestinal Fate, Microbial Transformation, Mechanistic Insights, and Prospects for Precision Nutrition

**DOI:** 10.3390/metabo16090680

**Published:** 2026-09-16

**Authors:** Lei Yang, Chaowei Wang, Liangyu Cui, Yongxiang Zhu, Xi Wang

**Affiliations:** 1 School of Nursing, Health Science Center, Xi’an Jiaotong University, Xi’an 710061, China; 2 Key Laboratory of Trace Elements and Endemic Diseases of National Health and Family Planning Commission, Health Science Center, School of Public Health, Xi’an Jiaotong University, Xi’an 710061, China; 3 Administrative Committee of Yunnan Baoshan Industial Park, Baoshan 678000, China; 4 Health Science Center, Department of Occupational and Environmental Health, School of Public Health, Xi’an Jiaotong University, Xi’an 710061, China

**Keywords:** coffee, gut microbiota, short-chain fatty acids, gut–liver axis, type 2 diabetes, precision nutrition

## Abstract

Coffee is one of the most widely consumed beverages worldwide and a major dietary source of bioactive compounds, including caffeine, chlorogenic acids (CGAs), trigonelline, non-digestible polysaccharides, and roasting-derived melanoidins. Habitual coffee consumption has been associated with a lower risk of type 2 diabetes (T2D) and more favorable liver-related outcomes, whereas evidence regarding obesity and metabolic dysfunction-associated steatotic liver disease (MASLD) remains heterogeneous. These associations may reflect interactions among coffee constituents, the gut microbiota, and host metabolic phenotypes rather than the effects of individual compounds alone. In this focused narrative review, we integrate evidence on the gastrointestinal fate, microbial transformation, and systemic availability of major coffee constituents, with particular emphasis on short-chain fatty acids, CGA-derived phenolic acids, and microbiota-dependent bile acid metabolism. We examine how these pathways may influence intestinal barrier integrity, mucosal immunity, enteroendocrine signaling, and gut–liver communication then consider their potential relevance to the regulation of energy, glucose, and lipid metabolism. We evaluate the relevance of these pathways to obesity, T2D, and MASLD, as well as major sources of inter-individual variability, including host genetics, baseline microbiota, metabolic phenotypes, background diet, and coffee composition, processing, brewing, filtration, dose, and beverage additives. Finally, we discuss physiologically relevant exposure levels, candidate biomarkers of individual responses, and risk–benefit considerations. Although mechanistic and preclinical evidence is accumulating, human studies directly demonstrating that coffee-induced microbiota changes mediate beneficial metabolic outcomes remain limited. Future randomized and mechanistic studies should integrate chemically characterized coffee exposures with longitudinal microbiome, metabolomic, physiological, and clinically relevant outcome measurements.

## 1. Introduction

Coffee is one of the most widely consumed beverages globally and is a major dietary source of caffeine and polyphenolic compounds [1]. Beyond caffeine, coffee contains chlorogenic acids (CGAs) and their derivatives, the alkaloid trigonelline, and diterpenes such as cafestol and kahweol [2,3,4]. Additionally, coffee beans are rich in cell-wall polysaccharides, primarily arabinogalactans and galactomannans, whereas Maillard and polymerization reactions during roasting generate structurally complex, high-molecular-weight melanoidins [5,6]. Accumulating evidence suggests that coffee or coffee-derived preparations can influence the gut microbial composition, microbial metabolites, hepatic inflammation, and metabolic phenotypes [7,8].

The physiological effects of coffee are partly determined by differences in the bioaccessibility and gastrointestinal fate of its constituents, which dictate where and to what extent they interact with the gut microbiota [9,10]. Whereas caffeine undergoes rapid absorption in the upper gastrointestinal tract and is subsequently metabolized primarily in the liver [11,12], substantial fractions of CGAs, ester-bound phenolic compounds, non-digestible polysaccharides, and melanoidin-associated compounds may escape digestion and absorption in the small intestine [10,13,14,15]. Colonic microbes not only ferment these complex glycans and high-molecular-weight roasting by-products to yield short-chain fatty acids (SCFAs), but also sequentially transform polyphenols into bioactive low-molecular-weight phenolic acids [10,13,14]. Concurrently, the gut microbiota modifies the host bile acid pool through diverse biotransformation reactions, including deconjugation, dehydroxylation, oxidation, and epimerization [16,17]. Together, these host–microbiome interactions provide a potential link between the gastrointestinal processing of coffee constituents and downstream effects on intestinal barrier function, mucosal immunity, and gut–liver signaling. Accordingly, the health effects of coffee may not be attributable to a single compound but may instead arise from interactions among coffee-derived inputs, the gut microbiome, and host metabolic networks.

In vitro and animal studies have suggested that coffee or selected coffee-derived preparations can alter the gut microbial composition and microbial metabolite profiles and may reduce hepatic lipid accumulation in some experimental models [7,8,18]. However, human clinical trials remain sparse, are often constrained by small sample sizes, and have produced inconsistent microbial and metabolic outcomes. Epidemiological cohorts associate habitual coffee consumption with a lower risk of type 2 diabetes (T2D) and more favorable liver-related outcomes [19,20,21], yet these observational associations do not establish causality. Indeed, the effects of coffee on insulin sensitivity, postprandial glycemic excursions, and hepatic steatosis vary across study designs and clinical populations [22,23,24,25]. This phenotypic variation is compounded by the chemical heterogeneity of the beverage itself, which is influenced by the bean species and cultivar, roasting degree, brewing conditions, and filtration method [2,3,4,5,6,26,27]. Inter-individual variation in host genetics, baseline microbiome composition, background diets, and metabolic states may further contribute to differences in the absorption, colonic transformation, and systemic physiological response to coffee intake [9,28,29,30].

Previous reviews have generally addressed coffee, the gut microbiota, gastrointestinal function, coffee-derived compounds, or metabolic outcomes from separate perspectives, leaving the evidence fragmented rather than organized within a continuous exposure-to-effect framework [31]. In particular, the gastrointestinal fate of coffee constituents, their colonic microbial transformation, host absorption, and systemic metabolic actions have not been comprehensively integrated. Moreover, the effects of roasting, brewing, filtration, the dose, the background diet, host genetics, the baseline microbiota, and metabolic phenotype have rarely been considered together as determinants of individualized responses. In this review, we address these gaps by integrating the gastrointestinal processing of coffee constituents with microbial metabolism, host exposure, and cardiometabolic outcomes. We distinguish rapidly absorbed compounds, such as caffeine, from non-digestible polysaccharides, melanoidins, and phenolic substrates that reach the colon and undergo microbial transformation and examine how the resulting short-chain fatty acids, chlorogenic acid-derived phenolic acids, and microbiota-dependent bile acid changes may influence intestinal barrier integrity, mucosal immunity, enteroendocrine signaling, and gut–liver communication. By evaluating evidence according to the study type and separating biological plausibility from demonstrated microbiota-mediated causality, we further develop a precision-nutrition framework that incorporates coffee processing, dose–exposure relationships, host genetics, baseline microbiota, the dietary context, and the metabolic phenotype. This framework may help explain inter-individual variability, identify candidate exposure and response biomarkers, and inform the design of future human studies.

### Literature Search and Evidence Synthesis

This article was designed as a focused narrative review rather than a systematic or scoping review. A non-systematic literature search was conducted in PubMed/MEDLINE/Web of Science from database inception and in Google Scholar through 1 July 2026. The search covered coffee and its major constituents in relation to the gut microbiota, microbial metabolites, intestinal barrier function, the gut–liver axis, metabolic and hepatic outcomes, coffee processing and brewing, and pharmacokinetics and exposure. Detailed search strings, eligibility criteria, and supplementary search procedures are provided in the Supplementary Materials. Records were assessed for relevance at the title and abstract level, with a full-text review performed when necessary; reference-list screening and citation tracking were also used to identify additional relevant studies. Human intervention studies, prospective cohort studies, and pharmacokinetic investigations were prioritized for conclusions concerning exposure, clinical relevance, and translational potential, whereas animal and in vitro studies were used primarily to evaluate mechanistic plausibility. Evidence from whole brewed coffee was distinguished from evidence involving isolated compounds, extracts, or other coffee-derived preparations. Publications based on the same cohort or experimental dataset were considered together and were not treated as independent replications. Studies were prioritized according to their direct relevance, methodological contribution, translational value, recency where appropriate, and mechanistic informativeness. L.Y. and C.W. conducted the initial relevance assessment and subsequent review of potentially relevant studies. Because this was a focused narrative review, the search and selection process was not intended to be exhaustive and may be subject to selection and publication bias. The detailed search and selection procedures are provided in the Supplementary Materials.

## 2. Bioactive Constituents of Coffee and Their Gastrointestinal Fate

**Figure 1:**
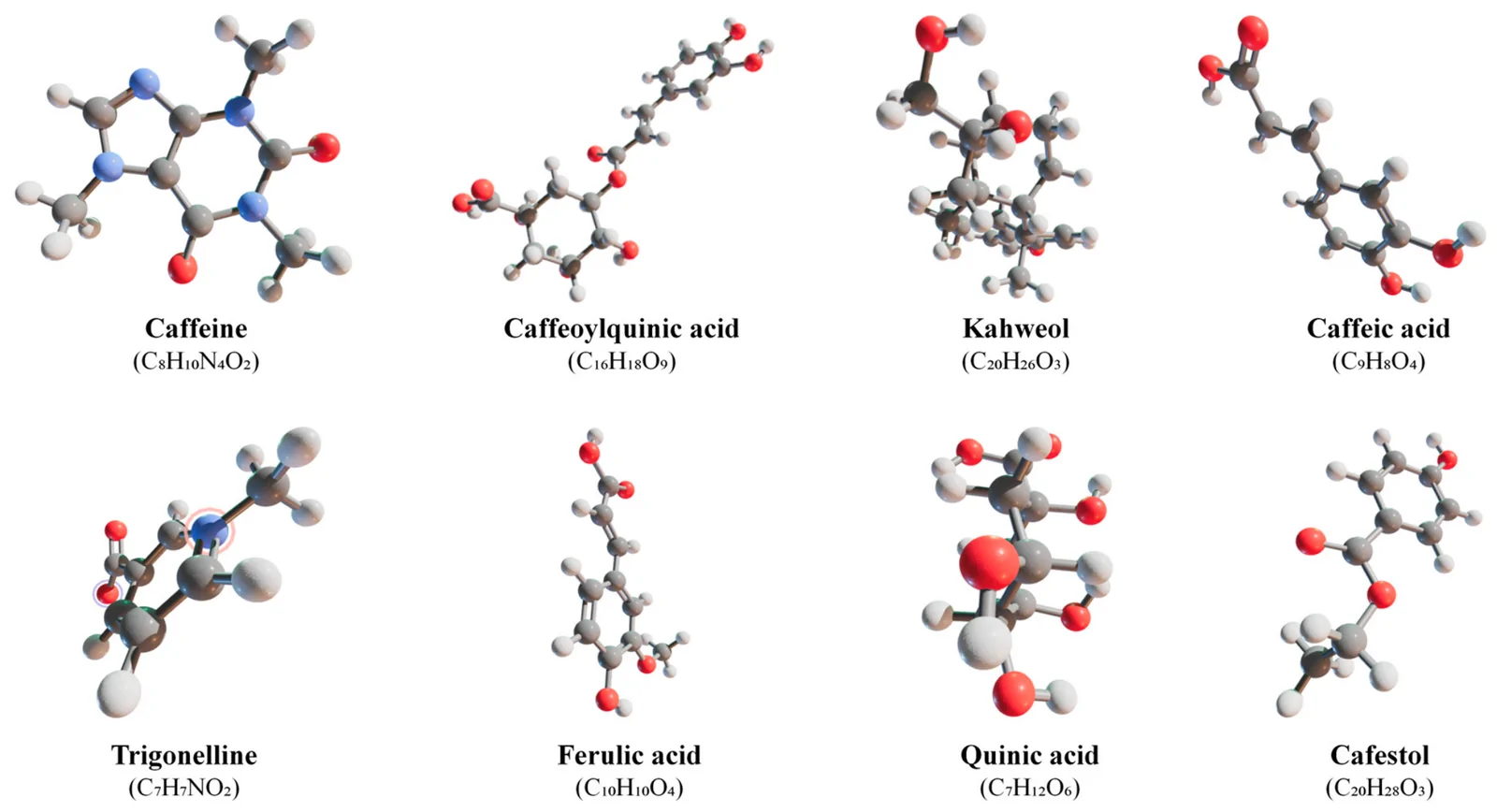
**Representative molecular structures of selected coffee constituents.** The structures shown represent the selected bioactive and matrix-associated constituents of coffee, including caffeine, chlorogenic-acid-related compounds, trigonelline, diterpenes, and roasting-derived compounds.

Coffee is a complex chemical matrix comprising caffeine, trigonelline, CGAs, non-digestible polysaccharides, soluble dietary fiber, macromolecular melanoidins, lipophilic diterpenes, and a diverse array of thermally generated low-molecular-weight roasting by-products. Owing to their distinct physicochemical properties and interactions within the food matrix, these constituents display highly divergent bioaccessibility, absorption kinetics, and metabolic fates along the gastrointestinal (GI) tract. Table 1 and Figure 1 summarize the molecular structures, GI fate, and putative physiological actions of these primary coffee bioactives.

### 2.1. Caffeine Absorption, Hepatic Metabolism, and Microbiota Interactions

Caffeine (1,3,7-trimethylxanthine), the principal methylxanthine alkaloid in coffee, is rapidly and efficiently absorbed after oral ingestion [11]. After absorption, caffeine is primarily metabolized in the liver by cytochrome P450 1A2 (CYP1A2) through sequential demethylation to form paraxanthine, theobromine, and theophylline [12]. Systemic CYP1A2 activity varies substantially among individuals, with genetic variation contributing to differences in caffeine clearance and systemic exposure [28]. Pharmacologically, caffeine acts as a competitive antagonist of adenosine receptors and modulates intracellular cyclic adenosine monophosphate (cAMP) signaling pathways [32].As a ubiquitous second messenger, cAMP can regulate epithelial tight-junction permeability and contribute to the maintenance of epithelial barrier integrity [33].

**Table 1:** Major coffee constituent classes, gastrointestinal fate, microbial and host metabolic pathways, and potential biological effects.

Major Constituent Class	Representative Compounds	Characteristics of Absorption	Major Metabolic Pathways	Potential Biological Effects
Caffeine	1,3,7-Trimethylxanthine (C_8_H_10_N_4_O_2_)	A small, weakly basic methylxanthine that is rapidly and almost completely absorbed after oral ingestion, resulting in limited exposure to intact caffeine in the colon [11]	Predominantly metabolized in the liver by CYP1A2 to paraxanthine, theobromine, and theophylline, followed by further metabolism and urinary excretion [12,34]	Caffeine acts primarily as an antagonist of adenosine receptors and may influence neural and metabolic signaling [32]. Its effects on the gut microbiota appear to be context-dependent and may involve changes in host metabolism, gastrointestinal function, or direct microbial exposure [7,35,36,37,38]
Trigonelline	*N*-Methylnicotinic acid (trigonelline; C_7_H_7_NO_2_)	A highly water-soluble betaine alkaloid containing a quaternary pyridinium group and a carboxylate moiety. Its concentration in coffee varies with coffee species and roasting conditions, and roasting can lead to its degradation into nicotinic acid and related products [3]	Partly degraded during roasting to form nicotinic acid and other products; after ingestion, the parent compound and its metabolites are eliminated in urine [3]	Trigonelline has been investigated for potential antioxidant, anti-inflammatory, glucose-regulatory, and lipid-regulatory activities, although the evidence is largely derived from experimental studies. Coffee extracts and selected coffee compounds can modulate the growth of some probiotic bacteria in vitro [39]
Chlorogenic acids	Caffeoylquinic, feruloylquinic and dicaffeoylquinic acids; 5-*O*-caffeoylquinic acid (5-CQA; C_16_H_18_O_9_) is the predominant representative	Polar esterified phenolic acids containing multiple hydroxyl groups and a quinic-acid moiety. Intact compounds may be partly absorbed or hydrolyzed in the upper gastrointestinal tract, whereas a proportion remains available for colonic microbial transformation [10,15,40,41,42,43,44]	Hydrolyzed by host or microbial esterases and further converted into caffeic acid, dihydrocaffeic acid, dihydroferulic acid, and other low-molecular-weight phenolic acids; absorbed metabolites subsequently undergo glucuronidation, sulfation, and methylation [9,14,15,40,41,42]	Chlorogenic acids and their microbial metabolites may influence gut microbial composition and metabolic signaling, with potential downstream effects on intestinal barrier function, metabolic endotoxemia, hepatic lipid accumulation, glucose homeostasis, inflammation, and oxidative stress [14,15,39,45,46,47,48,49,50]
Other phenolic compounds	Caffeic, ferulic, *p*-coumaric, protocatechuic and vanillic acids	Their gastrointestinal fate varies with molecular size, hydroxylation, methoxylation, ionization state, and matrix association. Free low-molecular-weight phenolic acids may be absorbed in the upper gastrointestinal tract, whereas more polar, conjugated or matrix-associated forms may undergo colonic transformation [10,40,41,42,43,44]	Released or transformed through host and microbial metabolism, followed by glucuronidation, sulfation, or methylation in the intestinal wall and liver [9,15,40,41,42]	Caffeic acid and related phenolic metabolites may influence oxidative stress, inflammation, and hepatic lipid metabolism, whereas evidence for direct regulation of epithelial barrier function and gut–liver signaling is mainly derived from studies of chlorogenic acids, coffee extracts, or experimental models [47,48,49,50,51,52,53,54]
Water-soluble polysaccharides	Arabinogalactans, galactomannans, and their degradation products	High-molecular-weight, hydrophilic coffee polysaccharides are poorly digested and absorbed intact. A proportion may reach the colon, where it can be fermented by the gut microbiota [13]	Partly fermented by the colonic microbiota, contributing to the production of acetate, propionate, and butyrate, as well as other microbial metabolites [7,13,14]	Provide substrates for microbial fermentation and may support epithelial barrier and metabolic homeostasis through SCFA-dependent pathways [13,51,55,56]
Insoluble dietary fiber	Cellulose, poorly soluble galactomannans, and residual cell-wall material	Insoluble coffee-derived fiber consists largely of structural polysaccharides and residual cell-wall material that resist small-intestinal digestion and absorption. Its fermentability in the colon depends on the polymer composition, particle structure, and physical accessibility; poorly fermentable fractions are largely excreted in feces [5,8,13,57]	Fermentable fractions may be utilized by the colonic microbiota, whereas poorly fermentable material is excreted in feces [8,13,57]	May provide fermentable substrates and influence microbial and metabolic profiles, although evidence is derived mainly from experimental studies of coffee by-products rather than conventionally brewed coffee [8,57]
Melanoidins	Heterogeneous nitrogen-containing macromolecules generated during roasting, comprising polysaccharide-derived, nitrogenous and phenolic moieties	Melanoidins are heterogeneous, high-molecular-weight Maillard-reaction products containing polysaccharide-derived, nitrogenous, and phenolic structures. Their intact absorption is expected to be limited, while fractions reaching the colon may undergo microbial transformation and release or generate lower-molecular-weight metabolites [6,13]	Partly fermented by intestinal microorganisms, with transformation or release of bound phenolic structures and generation of SCFAs and low-molecular-weight phenolic acids [6,13,14,15,44]	May exert dietary-fiber-like and microbiota-modulating effects through colonic fermentation, although their structures and biological activities vary with roasting and molecular compositions [6,13]
Diterpenes	Cafestol, kahweol, and their fatty acid esters	Lipophilic diterpenes, including cafestol and kahweol, commonly present as fatty-acid esters. Their beverage concentrations, and thus dietary exposure, are strongly determined by brewing and filtration; paper filtration substantially lowers concentrations relative to unfiltered preparations [4,58,59,60]	Their absorption and metabolism are influenced by their lipophilicity and esterification state, while their physiological effects involve pathways related to cholesterol and lipid homeostasis [4,58,60]	High exposure to cafestol and kahweol from unfiltered coffee can increase total and LDL-cholesterol concentrations [58,60]
Roasting-derived volatile compounds and other small molecules	Furans, pyrazines, aldehydes, ketones, sulfur-containing compounds, nicotinic acid, and *N*-methylpyridinium	This is a chemically heterogeneous group whose gastrointestinal fate is compound-specific and cannot be generalized reliably at the class level. Molecular size, polarity, volatility, ionization, and chemical stability are important determinants [26,27,61,62]	Metabolic pathways are compound-specific and cannot be generalized across this chemically heterogeneous group [26,27,61,62]	Volatile compounds generated during roasting are major contributors to coffee aroma and flavor. Selected non-volatile roasting products, particularly N-methylpyridinium, may affect hepatic de novo lipogenesis in experimental hepatocyte models [27,61,62,63]

Given that caffeine is not a typical substrate for colonic fermentation, its influence on the gut microbiota is likely indirect and might be mediated through changes in GI motility, neuroendocrine signaling, and the host systemic metabolic environment. Human cohort studies indicate that high caffeine consumption is associated with alterations in mucosal microbiota richness and the relative abundance of specific taxa, such as *Faecalibacterium* [35]. Furthermore, habitual coffee intake is associated with distinct fecal microbiome signatures, including a reproducible positive association with the prevalence and abundance of *Lawsonibacter asaccharolyticus* [29,36]. Preclinical studies in rodent models suggest that the administration of green coffee extract or purified caffeine modulates the expression of key tight-junction proteins, including occludin, claudins, and zonula occludens 1 (ZO-1) [37,64], although definitive evidence of a direct causal effect of caffeine on the intestinal epithelium remains limited. In mice with high-fat-diet-induced obesity, chronic caffeine supplementation altered the taxonomic composition of the gut microbiota and the serum metabolome and was accompanied by reductions in body weight gain, hyperglycemia, and dyslipidemia [37]. These findings suggest that caffeine can modify the host physiological environment and thereby indirectly affect the gut microbiota.

### 2.2. Microbial Biotransformation of Chlorogenic Acids

CGAs represent the predominant class of polyphenolic compounds in coffee, with 5-O-caffeoylquinic acid (5-CQA) serving as the major isomer [2]. Unlike caffeine, which is rapidly absorbed in the upper gut, a substantial fraction of ingested CGAs escapes absorption in the upper gastrointestinal tract and remains available for microbial transformation in the colon [9,10,15]. Whereas some CGAs undergo absorption and hydrolysis before reaching the colon, the remaining compounds or their metabolites can undergo further microbial transformation in the colon [10,15]. This hydrolysis is primarily mediated by esterase-producing bacteria including *Bifidobacterium* and *Eubacterium ramulus* [45,65]. Caffeic acid subsequently undergoes sequential microbial degradation through reduction, dehydroxylation, and decarboxylation pathways, yielding dihydrocaffeic acid, dihydroferulic acid, and an array of hydroxylated phenylpropionic, phenylacetic, and benzoic acid derivatives [14,15]. These low-molecular-weight microbial metabolites are absorbed across the colonic epithelium and undergo phase II conjugation, including glucuronidation, sulfation, and methylation, in the gut mucosa and liver before entering the systemic circulation [9,15].

In vitro human colonic fermentation models have demonstrated that coffee extracts and purified CGAs selectively modulate the growth of specific fecal bacterial taxa and promote the generation of diverse phenolic acids [14,15]. Consistent with these findings, human pharmacokinetic studies have detected coffee-derived phenolic acid metabolites, including benzoic acid- and hippuric acid-related metabolites, in plasma and urine following coffee ingestion [9]. These studies establish that CGA-related compounds can undergo microbial transformation and that their downstream metabolites can be absorbed after colonic generation.

### 2.3. Colonic Co-Fermentation of Dietary Fiber, Melanoidins, and Polyphenols

The cell-wall polysaccharide matrix of coffee beans primarily consists of arabinogalactans and galactomannans, alongside a smaller cellulose fraction [5,6]. During roasting, degradation and Maillard reactions involving sugars, amino compounds, and other coffee constituents generate high-molecular-weight melanoidins, while also altering the structure of coffee polysaccharides. With thermal processing, these structural glycans undergo complex Maillard reactions and polymerization events with CGA-derived products, generating high-molecular-weight melanoidins that enter the final beverage [5,6]. Some fractions of a substantial portion of these soluble polysaccharides, carbohydrate-associated melanoidin complexes, and bound phenolics resist digestion in the upper GI tract and reach the colon, although these components display marked differences in fermentability [6,13]. In the large intestine, arabinogalactans and galactomannans can undergo microbiota-mediated degradation by specialized carbohydrate-active enzymes (CAZymes), generating SCFAs, principally acetate, propionate, and butyrate [13,14]. Notably, the fermentation of arabinogalactan-rich coffee fractions by the colonic microbiota increases SCFA production and is accompanied by the formation of dihydrocaffeic and dihydroferulic acids [13,14]. Although melanoidin-rich fractions may influence colonic fermentation, whether they directly affect microbial secondary bile acid transformation remains unknown. These findings indicate that coffee-derived polysaccharides, melanoidins, and bound phenolic compounds can reach the colon and serve as substrates or modifiers of microbial metabolism.

### 2.4. Diterpenes, Trigonelline, and Processing-Dependent Chemical Heterogeneity

The lipophilic diterpenes cafestol and kahweol are present in the lipid fraction of coffee oil; consequently, their concentrations in the beverage depend strongly on the preparation method, with paper filtration substantially reducing their levels compared with boiling or other unfiltered methods, such as French press preparation [4]. The trigonelline concentration is determined by the coffee type and roasting degree; roasting induces the thermal degradation of trigonelline and contributes to the formation of nicotinic acid and various volatile aroma compounds [3,26,27]. In vitro studies indicate that coffee extracts and trigonelline can modulate the growth of potentially beneficial bacteria, including *Lactiplantibacillus plantarum*, *Lactobacillus acidophilus*, and *Bifidobacterium* spp. [39]. Furthermore, diet-induced obese rats fed coffee pulp, which is rich in trigonelline, CGAs, diterpenes, and fiber, exhibit marked shifts in the gut microbial composition that are associated with improvements in systemic glycemic and lipid profiles [57].

## 3. Effects of Coffee on Gut Microbiota Composition, Diversity, and Function

Current evidence suggests that coffee consumption is associated with selective shifts in the gut microbial composition and function, including the enrichment of SCFA-associated taxa, potential suppression of pathobionts, and alterations in microbial metabolic outputs. These responses appear to vary according to coffee composition, the baseline microbiota, and the host metabolic status, potentially contributing to inter-individual heterogeneity in the biological effects of coffee.

### 3.1. Changes in Potentially Beneficial Taxa and SCFA-Producing Communities

Human intervention and cohort studies and clinical evidence suggest that coffee consumption is associated with changes in selected microbial taxa, although the magnitude and consistency of these changes vary across populations and study designs [29,31,66,67]. In a small 3-week human intervention, daily coffee intake was accompanied by increased *Bifidobacterium*-related abundance and metabolic activity, without major changes in the overall community structure [66]. In a comparative study evaluating coffee and galacto-oligosaccharides, coffee consumption produced a similar bifidogenic response and altered selected clostridial and *Escherichia*-related measures [67]. *Bifidobacterium* species degrade complex dietary glycans to produce acetate and lactate, which serve as substrates for secondary fermenters through microbial cross-feeding networks and can ultimately promote butyrate synthesis [68]. These findings suggest that coffee may selectively influence certain microbial populations rather than broadly restructure the gut microbial community.

Human observational studies have associated higher caffeine intake with differences in mucosa-associated microbial richness and the relative abundance of selected taxa, including *Faecalibacterium* [35].

One of the most reproducible taxonomic signatures associated with coffee intake across geographically diverse cohorts is the enrichment of *L. asaccharolyticus*. Large-scale metagenomic association studies have identified coffee consumption as an important dietary predictor of both the prevalence and abundance of this species, a finding supported by in vitro culture experiments showing that it responds to coffee exposure [36]. Consequently, *L. asaccharolyticus* represents a candidate marker of coffee-associated microbial responses; however, its functional role in coffee-component metabolism or host metabolic regulation remains unclear.

Beyond caffeine, coffee constituents such as CGAs, arabinogalactans, and roasting-derived melanoidins might act together to shape the microbial ecosystem. In vitro studies further indicate that whole coffee, CGAs, polysaccharides, and melanoidins can selectively influence microbial growth and metabolite production [13,14,15]. These findings support the possibility that coffee constituents affect microbial function, in addition to the taxonomic composition. Nevertheless, the extent to which these responses occur after habitual coffee consumption in humans and whether they produce clinically relevant changes in SCFA or phenolic-acid metabolism remain uncertain.

### 3.2. Potential Suppression of Pathobionts and Inflammation-Associated Taxa

In addition to supporting potentially beneficial commensals, coffee and its bioactive derivatives might selectively inhibit some opportunistic pathogens and inflammation-associated taxa. In vitro assays indicate that coffee-derived phenolics disrupt bacterial cell membrane integrity, impair transmembrane proton gradients, and inhibit biofilm formation under specific experimental conditions [69,70]. Parent CGAs and their downstream microbial catabolites, such as dihydrocaffeic acid and hydroxyphenylpropionic acids, might further modify the microenvironment and alter competitive interactions in the colonic niche [14,15,69]. Under anaerobic fermentation conditions, coffee and CGAs can reduce the abundance of pathobionts such as *Clostridium perfringens* [14] and selectively inhibit the growth of specific *Enterobacteriaceae* members [70].

Human observational data provide partial support for these findings, with higher caffeine intake being associated with differences in the relative abundance of selected mucosa-associated taxa [35]. Human intervention studies have reported increased fecal *Bifidobacterium* abundance and changes in selected clostridial or *Escherichia*-related measures following coffee consumption [66,67]. In vitro fermentation studies have also reported changes in selected potentially harmful clostridia after exposure to coffee or CGAs [14]. Lipopolysaccharide (LPS) derived from Gram-negative bacteria can activate Toll-like receptor 4 (TLR4) signaling. When epithelial barrier dysfunction increases the entry of microbial products into the circulation, this process may contribute to low-grade inflammation and insulin resistance [46].

### 3.3. Effects of Compound Diversity and Host Pathophysiological Background

The microbial response to coffee is shaped by interactions between compound-specific effects and host physiological heterogeneity. Differences in the absorption, degradation, and fermentation of individual coffee constituents may lead to distinct microbial responses along the gastrointestinal tract [13,14,15]. A pilot randomized, placebo-controlled trial in patients with T2D and non-alcoholic fatty liver disease (NAFLD; historical terminology, now largely encompassed by MASLD) reported short-term changes in selected gut microbial features after supplementation with coffee components [38]. Compared with metabolically healthy individuals, patients with metabolic disorders may exhibit different baseline microbial configurations and microbial metabolic capacities. These differences could contribute to inter-individual variation in the direction and magnitude of coffee-associated microbial responses, although their clinical relevance remains uncertain [29,31,35,38].

## 4. Host Metabolic Regulation Through Coffee–Microbiota Interactions

**Figure 2:**
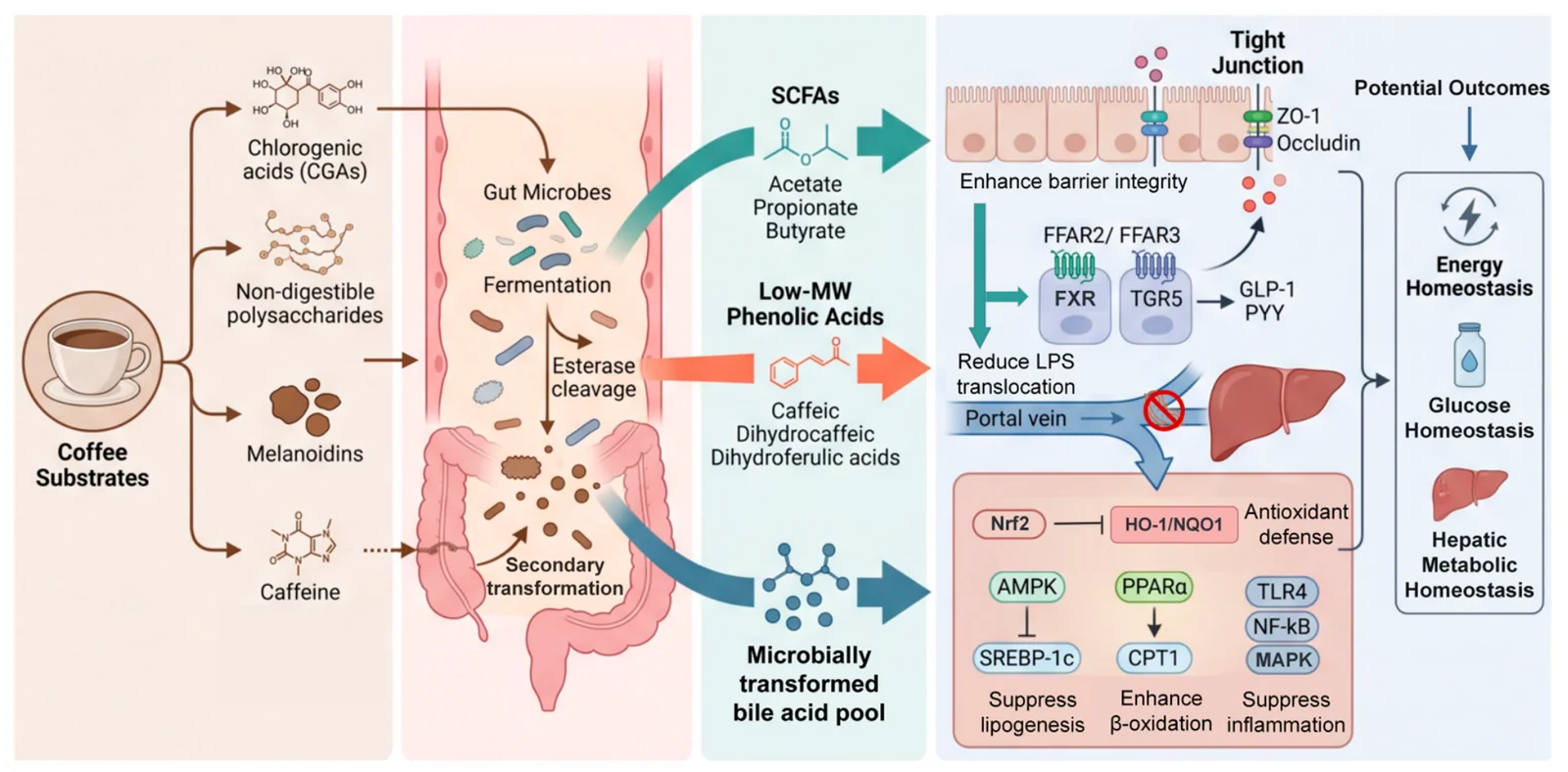
**Coffee–microbiota–host metabolic axis: gastrointestinal processing, microbial transformation, and systemic metabolic regulation.** Caffeine is rapidly absorbed, whereas chlorogenic acids (CGAs), non-digestible polysaccharides, and melanoidins reach the colon and serve as substrates for microbial fermentation and biotransformation. Gut microbiota generate short-chain fatty acids (SCFAs: acetate, propionate, butyrate) through polysaccharide fermentation, catalyze CGA esterase cleavage and sequential transformation into low-molecular-weight phenolic acids (caffeic, dihydrocaffeic, dihydroferulic acids), and modify the bile acid pool through deconjugation and dehydroxylation. These microbiota-derived metabolites, together with absorbed bioactives, influence host physiology. SCFAs enhance intestinal barrier integrity (tight-junction proteins ZO-1, occludin) and activate enteroendocrine FFAR2/FFAR3 receptors to stimulate GLP-1 and PYY secretion. Phenolic metabolites may reduce LPS translocation via portal circulation. In hepatocytes, coffee-related compounds modulate metabolic and inflammatory pathways: AMPK activation suppresses lipogenesis (SREBP-1c inhibition); PPARα activation enhances β-oxidation (CPT1); Nrf2 activation strengthens antioxidant defense (HO-1/NQO1); and TLR4/NF-κB/MAPK suppression attenuates inflammation. Microbially transformed bile acids activate FXR and TGR5, regulating bile acid homeostasis, glucose metabolism, and energy expenditure. Together, these pathways connect coffee substrates with energy homeostasis, glucose regulation, and hepatic metabolic health. AMPK, AMP-activated protein kinase; CPT1, carnitine palmitoyltransferase 1; FFAR2/3, free fatty acid receptor 2/3; FXR, farnesoid X receptor; GLP-1, glucagon-like peptide 1; HO-1, heme oxygenase 1; LPS, lipopolysaccharide; MAPK, mitogen-activated protein kinase; NF-κB, nuclear factor kappa B; NQO1, NAD(P)H quinone dehydrogenase 1; Nrf2, nuclear factor erythroid 2-related factor 2; PPARα, peroxisome proliferator-activated receptor alpha; PYY, peptide YY; SCFAs, short-chain fatty acids; SREBP-1c, sterol regulatory element-binding protein 1c; TGR5, G protein-coupled bile acid receptor 1; TLR4, Toll-like receptor 4; ZO-1, zonula occludens 1.

Coffee-derived substrates and their microbial metabolites may connect the intestinal lumen with systemic metabolism through partially interacting metabolic and signaling pathways. Among the candidate mediators, SCFAs and phenolic acids, together with potential alterations in microbial bile acid metabolism, may influence epithelial barrier function, enteroendocrine signaling, immune homeostasis, and gut–liver communication, thereby contributing to the regulation of energy, glucose, and lipid metabolism (Figure 2).

### 4.1. SCFA Generation, Epithelial Barrier Integrity, and Energy Homeostasis

Among microbially derived metabolites, SCFAs are plausible candidate mediators of the metabolic effects of coffee-derived components, because non-digestible polysaccharides and other high-molecular-weight coffee fractions can be fermented by human intestinal microbiota in vitro to generate acetate, propionate, and butyrate, while coffee-related components may also alter the gut microbiota and circulating SCFA profile in vivo [7,13,14]. Concurrently, free and bound polyphenols, such as CGAs, indirectly modulate SCFA profiles by altering microbial substrate preferences and community composition [7,14,15]. In animal models, interventions with coffee or selected coffee bioactives have sometimes produced parallel changes in gut microbiota composition, SCFA levels, and metabolic phenotypes [7,8,18,37].

SCFAs can regulate host metabolism through multiple pathways. Butyrate serves as an important energy source for colonocytes and supports epithelial barrier homeostasis through effects on oxidative metabolism, epithelial hypoxia, and tight-junction organization [51]. Short-chain fatty acids can activate free fatty acid receptor 2 (FFAR2) on enteroendocrine cells and stimulate GLP-1 secretion, with potential effects on appetite regulation and systemic energy metabolism [55].

### 4.2. Phenolic Acid Metabolites and Their Systemic Effects

As detailed in Section 2.2, microbial catabolism of CGAs and ester-bound phenolics can generate low-molecular-weight phenolic acids that can be absorbed after colonic formation and undergo phase II conjugation in the intestinal mucosa and liver [9,14,15,40,41]. Human studies have detected coffee-derived phenolic acid metabolites and their conjugates in plasma and urine after coffee ingestion, demonstrating systemic exposure to products of coffee-component metabolism [9,40,41,71]. However, the detection of these metabolites does not establish that they mediate the long-term metabolic associations observed in epidemiological studies.

Cell and animal studies have implicated chlorogenic acid, caffeic acid, and related coffee-derived phenolic compounds in redox, inflammatory, and metabolic signaling, including NF-κB/MAPK, Nrf2, and AMPK-related pathways. These phenolic acids also modulate mitogen-activated protein kinase (MAPK) pathways, including p38, JNK, and ERK, and promote the nuclear translocation of nuclear factor erythroid 2-related factor 2 (Nrf2) and the subsequent transcription of cytoprotective genes, including heme oxygenase 1 (HO-1), NAD(P)H quinone dehydrogenase 1 (NQO1), and glutathione-related antioxidant enzymes [47,48,52,53]. These findings provide a mechanistic basis for the possible effects on hepatic lipid metabolism, inflammation, and insulin sensitivity. Nevertheless, much of the evidence comes from cell culture and animal models using parent compounds or purified extracts at concentrations or doses that may not reflect the conjugated metabolites and exposure levels achieved after habitual coffee consumption in humans.

### 4.3. Microbial Bile Acid Transformation Pathways

Microbially mediated transformation of the bile acid pool represents another potential mechanism linking coffee intake to regulation of the gut–liver axis, although current evidence is predominantly limited to taxonomic associations and changes in specific bile acid profiles. High-molecular-weight coffee fractions can be fermented by human intestinal microbiota to produce acetate, propionate, and butyrate in vitro [13]. Whether this process alters the bile acid pool in vivo remains to be established. Preclinical interventions with caffeine or CGAs have also been reported to alter the intestinal microbial composition and the serum metabolome, including aspects of host bile acid metabolism [7,18,37].

The resident microbiota modifies the bile acid pool through deconjugation, dehydroxylation, oxidation, and epimerization reactions, converting primary bile acids into secondary species [16,72]. These metabolites function as endogenous ligands for farnesoid X receptor (FXR) and G protein-coupled bile acid receptor 1 (TGR5). Intestinal FXR activation promotes the secretion of FGF15 in mice, with the orthologous FGF19 pathway operating in humans, thereby contributing to the feedback regulation of hepatic bile acid synthesis [73,74]. TGR5 activation modulates GLP-1 release, immune-cell function, and energy expenditure [75]. However, clinical studies directly demonstrating that coffee modulates human metabolic outcomes through specific microbial taxa and defined bile acid-receptor pathways remain sparse.

### 4.4. Epithelial Barrier Function and the Gut–Liver Axis

The intestinal epithelial barrier constitutes an anatomical and immunological interface between the gut microbiota and host metabolism. It comprises the mucus layer, apical junctional complexes, mucosal immune cells, and the resident microbiota, which collectively regulate the translocation of pathogen-associated molecular patterns (PAMPs) and gut-derived metabolites into the portal circulation [51,76]. When barrier integrity is impaired, the increased translocation of Gram-negative bacterial lipopolysaccharide (LPS) may expose the liver to higher concentrations of microbial products [46,51,76]. LPS recognition through the LBP/CD14–TLR4/MD-2 system activates downstream NF-κB and MAPK-related pathways in innate immune cells [46,77].

The potential relevance of coffee to the epithelial barrier function is inferred from the known actions of SCFAs and phenolic metabolites on colonocytes, epithelial junctions, and mucosal immune signaling. Butyrate can support colonocyte energy metabolism and tight-junction function [51,56], whereas coffee-derived phenolic compounds may influence local redox balance and inflammatory pathways in experimental models [47,52]. For instance, butyrate can support epithelial homeostasis through effects on colonocyte oxidative metabolism, epithelial hypoxia/HIF signaling, and energy-regulatory pathways [51,56]. Nevertheless, consistent clinical evidence that coffee directly improves human epithelial barrier function through increased butyrate production or microbiome remodeling is lacking.

Preclinical studies indicate that coffee or selected coffee bioactives can alter the gut microbial composition and SCFA-related measurements in diet-induced metabolic models [7,8,18,37]. Other experimental studies suggest that coffee extracts or related bioactives may improve selected markers of intestinal barrier function and hepatic inflammation or lipid metabolism [53,64]. These findings support a possible connection between coffee-related microbial responses and hepatic physiology, but they do not establish that microbiome remodeling or increased SCFA production is necessary for the observed hepatic effects.

## 5. Potential Microbiome-Related Links Among Coffee Intake, Obesity, and Energy Homeostasis

Coffee may influence energy homeostasis predominantly through direct effects on host metabolic pathways, while microbiome-mediated mechanisms remain plausible but insufficiently established. The combined actions of caffeine, chlorogenic acids (CGAs), and microbiota-derived metabolites may further modulate energy intake and appetite regulation.

### 5.1. Regulation of Appetite and Enteroendocrine Gut–Brain Signaling

Human experimental studies indicate that caffeine intake can acutely stimulate thermogenesis and increase energy expenditure [78]. In a human experimental study, coffee and its constituent compounds also modified gastrointestinal hormone secretion and postprandial glucose tolerance [25,79]. These acute physiological effects should be distinguished from sustained reductions in body weight or adiposity. Experimental and human studies have reported that CGAs or coffee constituents can influence glucose handling and metabolic signaling, including AMPK-related pathways, and may attenuate postprandial glycemic responses [25,48]. These findings provide a possible basis for effects on appetite and energy balance, although they do not by themselves establish a sustained anti-obesity effect in humans.

The gut microbiota may provide an additional link between non-digestible coffee components and host satiety signaling. Building on the evidence that fermentable coffee-derived substrates can contribute to microbial SCFA production, SCFAs may serve as functional mediators between microbiome activity and enteroendocrine responses. Short-chain fatty acids can activate free fatty acid receptor-dependent signaling in enteroendocrine cells, with the FFAR2-mediated stimulation of GLP-1 secretion providing a plausible mechanism for effects on satiety and energy regulation [55]. A human intervention study evaluating the targeted delivery of propionate to the colon supports the physiological relevance of SCFA signaling, but does not directly test coffee consumption [80]. In a small clinical trial, green coffee bean extract was associated with modest reductions in body weight and improvements in body composition; however, the concentrated amounts of CGAs and caffeine in this preparation differ from habitual coffee consumption [81]. Large prospective cohort studies have associated increases in coffee consumption without added sugar with less long-term weight gain [82]. In animal models, caffeine and CGA interventions have been associated with reductions in diet-induced weight gain or adiposity, whereas coffee-related interventions have also been linked to changes in gut microbial and metabolomic profiles [7,37,50]. Together, these findings support a potential microbiome-related contribution, although the relative importance of this pathway in humans remains to be established.

### 5.2. Promotion of Thermogenesis and Energy Expenditure

Caffeine can increase energy expenditure through direct host-mediated mechanisms. By antagonizing adenosine receptors, caffeine enhances sympathetic-catecholamine signaling, promotes lipolysis in white adipose tissue, and can increase lipid oxidation and energy expenditure [32,78,83]. Coffee-related bioactives can influence adipocyte signaling pathways involved in thermogenesis and metabolic regulation, including AMPK- and PPAR-related pathways [84,85]. In adipocytes undergoing browning, these signaling pathways are associated with the regulation of thermogenic and mitochondrial biogenesis programs, including PGC-1α and UCP1 [84,85].

Human experimental studies support acute caffeine-related increases in energy expenditure, and individuals with higher brown adipose tissue activity may show a greater thermogenic response to caffeine supplementation [78,83]. Cellular and animal studies indicate that caffeine and selected coffee-derived bioactives can induce adipose-tissue browning and alter selected thermogenic markers, including UCP1 and PGC-1α-related pathways [84,85]. These findings support a possible role for coffee constituents in adipose-tissue thermogenesis, but the contribution of microbial metabolites to this process has not been established in humans.

## 6. Coffee Consumption, T2D Risk, and Glucose Homeostasis

Coffee consumption is associated with a lower risk of type 2 diabetes (T2D), although the underlying mechanisms remain incompletely understood. This section summarizes the effects of coffee and its bioactive constituents on glucose and insulin homeostasis and evaluates the potential contribution of gut microbiota-related pathways to T2D and its systemic complications.

### 6.1. Postprandial Glycemic Responses and Insulin Homeostasis

In contrast to these potential effects of non-caffeine bioactives, acute human experimental studies show that caffeine exposure can transiently impair glucose tolerance [86]. By antagonizing adenosine receptors, caffeine may influence sympathetic and catecholaminergic signaling and can acutely reduce insulin sensitivity in humans [32,86]. Consequently, acute caffeine administration can temporarily decrease insulin sensitivity and may increase postprandial glucose responses [86]. In contrast, large prospective cohort studies have associated increases in coffee consumption with a lower subsequent risk of T2D [19]. Randomized clinical trials provide a more mixed picture: coffee consumption did not produce clear improvements in insulin sensitivity or established biological risk factors for T2D in one placebo-controlled trial [22], while another trial found effects on selected biological risk factors for T2D [24]. Notably, in patients with T2D and MASLD, randomized supplementation with caffeine and/or chlorogenic acid was not superior to placebo for most hepatic, metabolic, and inflammatory outcomes [23]. These findings indicate that the inverse associations observed in cohorts should not be interpreted as proof that caffeine, CGAs, or the gut microbiota directly improve glycemic control in patients with established metabolic disease. Cellular and animal studies suggest that chlorogenic acid and other coffee-derived bioactives may influence oxidative stress, inflammatory signaling, hepatic lipid and glucose metabolism, and insulin-related pathways [47,48,52,53]. These findings provide biological plausibility but do not demonstrate clinical efficacy in people with established T2D.

The gut microbiota represents a potential mediator linking habitual coffee intake with long-term changes in glucose metabolism. Human studies have reported associations between coffee consumption and gut microbial composition, including changes in selected taxa [29,35,36,66,67], while human pharmacokinetic studies confirm systemic exposure to coffee-derived phenolic acid metabolites after coffee intake [9,40,41]. In vitro and animal studies suggest that the microbial metabolism of non-digestible coffee fractions may influence SCFA production [7,13,14,15]. SCFAs and related microbial signals can affect enteroendocrine signaling, epithelial barrier function, and metabolic pathways [51,55,56]. However, no controlled human study has yet demonstrated that coffee-induced changes in microbial composition or microbial metabolites mediate improvements in insulin sensitivity, glycemic control, or T2D risk. These microbiome-related pathways should therefore be regarded as biologically plausible hypotheses.

### 6.2. Potential Implications for T2D-Related Systemic Complications

Diabetic complications and associated end-organ damage are driven by chronic hyperglycemia, insulin resistance, dyslipidemia, oxidative stress, and low-grade chronic inflammation [87]. Observational studies have linked gut microbiota-derived SCFA alterations with diabetic kidney disease and related metabolic complications [88].

Experimental studies suggest that selected coffee-derived compounds may influence oxidative stress, inflammatory signaling, and metabolic pathways relevant to hepatic and systemic complications [47,48,49,52,53]. In addition, mechanisms involving epithelial barrier integrity, microbial products such as LPS, and gut-derived metabolites are biologically relevant to systemic inflammation and metabolic complications [46,51,56,73,74,75]. However, direct clinical evidence for these microbiota-mediated protective effects remains limited.

## 7. Effects of Coffee on MASLD

Coffee intake has been associated with more favorable MASLD-related outcomes and may influence pathways involved in disease progression. Coffee bioactives and microbial metabolites may reduce hepatic steatosis, oxidative stress, and inflammation by activating AMPK and Nrf2, while suppressing SREBP-1c and TLR4/NF-κB signaling, thereby limiting hepatic stellate cell activation and fibrogenesis.

### 7.1. Hepatic Steatosis and Inflammatory Signaling

Human evidence regarding coffee and hepatic steatosis remains limited and heterogeneous. Observational studies have associated habitual coffee consumption with more favorable liver-related phenotypes [20,21], but such associations may be affected by differences in diet, adiposity, physical activity, alcohol intake, smoking, medication use, and other lifestyle factors. However, in patients with T2D and MASLD, randomized supplementation with caffeine and/or chlorogenic acid was not superior to a placebo for hepatic fat, liver stiffness, or most hepatic, metabolic, and inflammatory outcomes [23].

Hepatic steatosis is influenced by the balance among circulating fatty acid influx, de novo lipogenesis, fatty acid β-oxidation, and very-low-density lipoprotein secretion [89]. Cellular and animal studies suggest that selected coffee-derived bioactives, particularly chlorogenic acid and N-methylpyridinium, may influence AMPK-related lipid regulation and de novo lipogenesis, with effects involving ACC-related pathways [48,50,53,63]. ACC1 and ACC2 are important regulators of fatty acid synthesis and lipid homeostasis, and their phosphorylation status can influence hepatic lipid metabolism and insulin sensitivity [90]. Caffeine, trigonelline, melanoidins, and roasting-derived compounds may also contribute to experimental effects on lipid metabolism [48,50,53,63,78,84,85].

In experimental models, lipotoxic stress promotes mitochondrial dysfunction, reactive oxygen species generation, endoplasmic reticulum stress, hepatocyte injury, and inflammatory activation involving Kupffer cells and infiltrating macrophages [53,91]. Cell and animal studies indicate that caffeic acid and related coffee phenolics can modulate Nrf2-mediated cytoprotective pathways and may reduce the activation of TLR4–MyD88–NF-κB-related inflammatory signaling [49,52]. However, these studies often use parent compounds or purified extracts at concentrations and exposure patterns that may not reflect circulating conjugated metabolites or tissue exposure after habitual coffee consumption.

### 7.2. Liver Fibrosis and Disease Progression

Hepatic fibrosis is a major predictor of mortality and adverse clinical outcomes in MASLD, and persistent activation of hepatic stellate cells (HSCs) is central to fibrogenesis [92,93]. Lipotoxic hepatocyte injury, oxidative stress, and chronic inflammation promote the release of profibrotic cytokines, particularly transforming growth factor-β (TGF-β). This cytokine activates the TGF-β-Smad signaling pathway, promoting the transdifferentiation of quiescent HSCs into proliferative, contractile myofibroblast-like cells [54,92]. Preclinical studies indicate that chlorogenic acid can modulate profibrotic pathways and reduce α-SMA and type I collagen expression in experimental models [49,54]. In experimental studies, caffeine might also inhibit HSC proliferation and activation by modulating adenosine receptor-mediated signaling [94]. Additionally, observational studies in patients with fatty liver disease have consistently associated habitual coffee consumption with a lower likelihood of significant or advanced hepatic fibrosis, particularly among those with moderate-to-high intake [95,96]. Similar inverse associations have also been observed across other chronic liver disease populations [21,93]. However, these findings are predominantly observational and remain susceptible to residual confounding by overall dietary quality, smoking, alcohol intake, physical activity, and socioeconomic factors.

## 8. Inter-Individual Responses to Coffee: Genetic, Microbial, and Dietary Determinants

**Figure 3:**
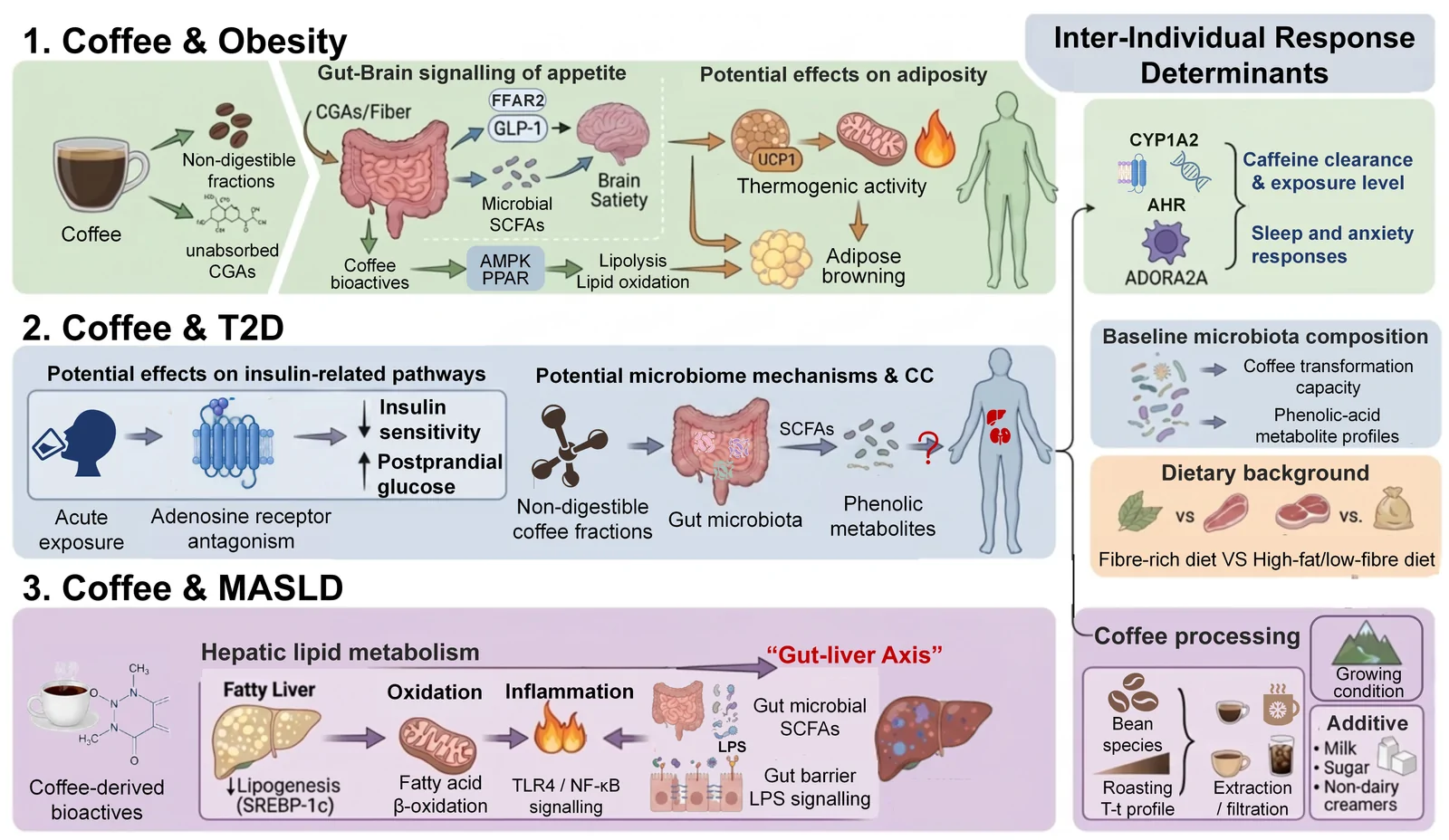
**Inter-individual determinants of metabolic responses to coffee in obesity, type 2 diabetes, and metabolic-dysfunction-associated steatotic liver disease.** Coffee constituents undergo differential gastrointestinal processing and microbial transformation, leading to diverse metabolic effects across three major cardiometabolic conditions. (**Left panels**) Proposed mechanisms linking coffee intake with (1) energy homeostasis and adiposity through gut-brain appetite signaling (FFAR2-mediated GLP-1 secretion), thermogenic activation (UCP1-mediated energy expenditure), and adipose browning (AMPK- and PPAR-dependent pathways); (2) glucose metabolism and insulin sensitivity, with acute caffeine-induced adenosine receptor antagonism transiently impairing insulin sensitivity, while non-digestible coffee fractions may support long-term glycemic regulation through microbiota-dependent SCFA production and phenolic metabolite generation; and (3) hepatic lipid metabolism and inflammation, in which coffee bioactives may attenuate hepatic steatosis through the AMPK-mediated suppression of de novo lipogenesis (SREBP-1c pathway), reduce oxidative stress and inflammation via TLR4/NF-κB pathway inhibition, and potentially limit fibrogenic progression through the modulation of hepatic stellate cell activation. Red question marks indicate pathways where direct evidence for microbiome-mediated causality in humans remains limited. (**Right panel**) Inter-individual response determinants include host genetics (CYP1A2, AHR, ADORA2A variants influencing caffeine metabolism, clearance, and physiological sensitivity), baseline gut microbiota composition (affecting chlorogenic acid biotransformation capacity and phenolic acid metabolite profiles), dietary background (fiber-rich vs. high-fat/low-fiber dietary patterns modulating microbial ecology and metabolic context), and coffee processing variables (bean species, roasting temperature-time profile, brewing/extraction method, filtration, and beverage additives such as milk, sugar, or non-dairy creamers).

As discussed in the preceding sections, coffee consumption is associated with diverse metabolic outcomes, including effects on energy balance (Section 5), glucose homeostasis and type 2 diabetes risk (Section 6), and hepatic steatosis and fibrosis (Section 7). However, these associations vary substantially across individuals. This inter-individual heterogeneity may arise from interactions among the host genetic background, baseline gut microbiota, metabolic phenotype, background diet, and coffee processing (Figure 3). Together, these factors may modify the direction and magnitude of metabolic responses to coffee.

### 8.1. Host Genetics, Caffeine Pharmacokinetics, and Physiological Responses

Caffeine clearance and physiological responses display substantial inter-individual variation, partly owing to host genetic differences [28,97,98,99,100,101,102]. Following gastrointestinal absorption, caffeine undergoes extensive hepatic oxidation, primarily catalyzed by CYP1A2 [11,12,28]. Genetic variants that modify CYP1A2 expression or enzymatic activity influence the systemic exposure to caffeine and its primary methylxanthine metabolites [12,28,99].

The CYP1A2 single-nucleotide polymorphism rs762551 is frequently studied in relation to caffeine metabolism. The AA genotype is often classified as a relatively fast-metabolizer genotype, whereas the AC and CC genotypes are commonly classified as relatively slow-metabolizer genotypes; however, this categorization may not consistently predict in vivo caffeine clearance across populations and exposure contexts [28,97,98,99]. These pharmacogenomic differences might modify cardiovascular and renal responses to coffee [97,98,99]. Some epidemiological studies reported genotype-dependent associations between coffee or caffeine intake and hypertension, kidney dysfunction, or cardiovascular outcomes, although the direction and consistency of these interactions vary across studies [97,98,99]. This difference might reflect the longer systemic exposure to caffeine in slow metabolizers, which prolongs adenosine receptor blockade and sympathetic activation [28,32]. However, clinical association studies remain inconsistent, with some cohorts reporting different gene–diet interactions [97,98,99]. Thus, CYP1A2 rs762551 genotyping alone is insufficient to predict individual cardiometabolic responses to coffee consumption.

Beyond CYP1A2, functional variants in the aryl hydrocarbon receptor gene (AHR) and adenosine A2A receptor gene (ADORA2A) might modulate caffeine clearance or physiological sensitivity [28,100,101]. The AHR protein is a transcription factor that regulates CYP1A2 expression; loci near AHR and CYP1A2 have been associated with caffeine metabolism and habitual coffee consumption in genome-wide association studies (GWAS) [28]. ADORA2A is an important receptor target of caffeine in the central nervous system, and variations in ADORA2A might influence susceptibility to caffeine-related sleep disruption and anxiety [32,100,101]. Genetic variation in bitter-taste receptor genes (TAS2R) and reward-related pathways might also affect flavor preference and habitual coffee intake [102].

### 8.2. Baseline Microbiota, Host Metabolic Phenotype, and Background Diet

The baseline gut microbiome profile is an important determinant of coffee biotransformation and physiological responses [13,15,29]. Differences in the microbiome composition, functional gene profiles, and metabolic capacity can influence the colonic degradation of CGAs, complex polysaccharides, and melanoidins, producing personalized metabolic profiles even under identical intake conditions [10,13,15,29]. Variations in microbial enzymatic activities involved in ester hydrolysis and carbohydrate degradation may shape the yield, kinetics, and availability of low-molecular-weight phenolic acids and SCFAs [13,14,15].

Although human and in vitro studies have reported changes in *Bifidobacterium*-related measures and other taxa after coffee exposure, the magnitude of these responses may depend on baseline community structure and ecological context [29,39,66,67]. A low baseline abundance does not necessarily indicate a poor response; individuals with a low initial abundance might show a greater relative increase owing to a greater ecological opportunity for expansion [29,30].

The host metabolic phenotype further shapes these microbial responses. Obesity, T2D, and MASLD are often accompanied by alterations in insulin sensitivity, inflammatory tone, gut microbial ecology, bile-acid signaling, and epithelial-barrier-related processes [18,23,38,46,64,72,73,74,75,76]. Consequently, metabolically compromised individuals might show different microbial and metabolic responses to coffee components than healthy individuals, although whether these differences increase or reduce potential benefits remains unresolved [18,23,38,64].

The background diet is also an important modifying factor [29,103]. Coffee polysaccharides and polyphenols enter a complex colonic network in which they interact with dietary fiber, other plant-derived polyphenols, lipids, and protein-derived substrates [13,29,39,103]. A fiber-rich dietary pattern can support carbohydrate-fermenting communities and cross-feeding networks, whereas high-fat or low-fiber dietary patterns may alter microbial ecology, bile-acid signaling, and epithelial-barrier-related processes [46,68,72,74,76,103].

### 8.3. Coffee Processing and Brewing as Determinants of Microbiome Responses

Coffee processing should be considered a sequence of matrix-transforming events rather than a simple change in the roast degree or the concentration of individual compounds. The bean species, cultivar, and geographical origin contribute to the initial chemical composition of green coffee, including the relative abundance of caffeine, CGAs, trigonelline, sucrose, cell-wall polysaccharides, proteins, lipids, and phenolic precursors [2,3,5,27,43,61]. For example, *Coffea arabica* and *Coffea canephora* may differ in their baseline concentrations of caffeine, CGAs, trigonelline, sucrose, and cell-wall polysaccharides, thereby providing different substrate pools for subsequent thermal reactions [2,3,39]. Roasting is further determined by the time-temperature profile, including the heating rate, maximum temperature, residence time, moisture loss, internal heat transfer, and cooling conditions [5,6,27,43,61]. These parameters regulate cell-wall disruption, water evaporation, polysaccharide depolymerization and rearrangement, CGA isomerization and lactonization, trigonelline degradation, lipid migration, Maillard reactions, caramelization, and polymerization [5,6,27,43,61]. Moderate heating and cell-wall disruption may increase the release and extractability of soluble arabinogalactan and galactomannan fractions, whereas prolonged or more severe heating may promote dehydration, the cleavage or rearrangement of glycosidic linkages, changes in molecular weight and branching, and condensation or cross-linking with other matrix components [5,6]. During the later stages of roasting, reducing sugars and sugar-derived carbonyl compounds react with amino groups from proteins, peptides, and amino acids; subsequent dehydration, fragmentation, Strecker degradation, condensation, and polymerization generate heterogeneous nitrogen-containing products collectively referred to as melanoidins [6,27,43]. These melanoidins may contain carbohydrate-, protein- or peptide-, and phenolic-associated structures that differ in molecular weight, polarity, redox activity, solubility, and susceptibility to hydrolysis [6,13,44,62]. Phenolic compounds may be physically entrapped within, non-covalently associated with, or incorporated into roasting-derived macromolecular fractions [6,10,44,62]. Consequently, coffees with a similar final color may still differ in the soluble fiber structure, melanoidin composition, and phenolic distribution when produced using different roasting curves.

CGAs are particularly sensitive to roast severity. With increasing thermal exposure, caffeoylquinic acids may undergo isomerization and other thermal transformations, with a reduction in intact parent CGAs and changes in CGA-related products [2,5,6,27,43,61]. In fact, increased roast severity generally decreases the proportion of intact parent CGAs and alters the relative distribution of CGA-related thermal and polymer-associated products [2,5,6,27,43,61]. These chemically distinct products may differ in gastrointestinal stability, microbial accessibility, and colonic fermentability [10,13,14,15,44,62,104]. Brewing further modifies the delivered exposure through the water temperature, contact time, pressure, particle size, coffee-to-water ratio, agitation, and filtration, thereby influencing the extraction of caffeine, soluble CGAs, trigonelline, soluble polysaccharides, melanoidin-associated compounds, lipids, and suspended particles [4,10,26,59,61]. Espresso, drip-filtered, French press, boiled, cold-brew, and instant coffee may therefore provide different proportions of rapidly absorbed compounds and digestion-resistant material to the colon [10,26,59,61]. From a microbiome perspective, these differences may alter the amount and chemical form of coffee-derived substrates available for microbial transformation, including the conversion of CGA-related compounds into lower-molecular-weight phenolic acids and the fermentation of resistant carbohydrate fractions with possible effects on short-chain fatty acid production [10,13,14,15,44]. However, most evidence concerning processing-dependent changes in the fiber structure, melanoidin composition, CGA bioaccessibility, and colonic fermentability is derived from chemical characterization, in vitro digestion, or ex vivo fermentation studies. These findings provide a mechanistic rationale for possible differences in microbial metabolism but do not yet establish that a specific bean species, roasting curve, or brewing method produces a reproducible microbiome or clinical benefit in humans.

The dose, frequency, and duration of coffee consumption, as well as the presence or absence of caffeine, might determine the magnitude and stability of microbial adaptation. Acute single-dose exposures alter transient metabolite fluxes but are unlikely to stably restructure the taxonomic composition of the microbiota [13,14,15,29,66,67]. By contrast, chronic daily intake provides repeated exposure to unabsorbed CGAs, melanoidins, and glycans, potentially producing sustained microbial metabolic adaptation [13,14,29,33,66,67]. In vitro fermentation studies and small human studies have reported the enrichment of *Bifidobacterium* and other potentially functional taxa after regular intake, whereas the overall community structure remains relatively stable [14,29,39,66,67]. These findings suggest that the microbial effects of coffee might first appear as changes in specific taxa or metabolic pathways rather than as extensive taxonomic restructuring. Caffeinated coffee might also indirectly modulate the microbiome by altering GI motility, neuroendocrine signaling, and luminal secretion [25,32]. Decaffeinated coffee retains non-caffeine constituents, including CGAs, melanoidins, and soluble polysaccharide fractions, and may therefore produce microbiome-related effects despite its lower caffeine content [6,13,39].

Adding dairy products, non-dairy creamers, refined sugar, or non-nutritive sweeteners alters the physicochemical and nutritional matrix of coffee and complicates the attribution of microbial responses to coffee alone. Milk proteins can interact physically or chemically with coffee polyphenols and might alter their absorption kinetics [105]. Human studies indicate that a nondairy creamer, but not milk, can delay the appearance of coffee-derived phenolic acid equivalents in plasma; the effects of different beverage matrices on colonic polyphenol delivery require further investigation [105]. Refined sugar and high-fat creamers increase the energy density of coffee beverages and may confound associations attributed to coffee itself [82,105]. Non-nutritive sweeteners can produce individualized microbiome and glucose-tolerance responses, whereas the dietary emulsifier carboxymethylcellulose has been shown, under controlled feeding conditions, to alter the gut microbiota and intestinal metabolome [106,107].

## 9. Translational Considerations for Precision Coffee Nutrition

Translating current evidence into precision coffee nutrition requires moving beyond mechanistic plausibility and population-level associations toward evidence that is physiologically relevant, individually predictive, and clinically actionable. This transition depends on three interrelated considerations: whether experimental doses reproduce attainable human exposures, whether biomarkers can reliably capture exposure and response heterogeneity, and whether potential benefits remain favorable after accounting for individual susceptibility, beverage preparation, and safety.

### 9.1. Dose-Exposure Relationships and Physiological Relevance

The coffee composition varies with the species or cultivar, roasting degree, brewing method, and filtration [2,3,4,5,6,26,43,59,61]. Biological exposure is further influenced by the serving volume, consumption frequency, and beverage matrix [9,71,105]. Consequently, cups per day provide only an approximate measure of biological exposure, particularly when the coffee composition and preparation method vary [9,71]. This distinction is particularly important for caffeine and chlorogenic acids because of their different gastrointestinal fates [11,13,14,15,40,41,42,44,104].

Caffeine is rapidly absorbed in the upper gastrointestinal tract and subsequently metabolized mainly into paraxanthine, theobromine, and theophylline [11,12,28,34]. In regular coffee consumers, circulating concentrations of caffeine and its primary metabolites depend on the dose, sampling time, repeated exposure, and individual metabolic capacity [11,12,28,34]. Under a higher single-dose condition of 7.5 mg/kg, plasma concentrations of paraxanthine, theobromine, and theophylline remained relatively stable for at least 10 h in a human pharmacokinetic study, indicating prolonged metabolic availability [34]. By contrast, the bioavailability of chlorogenic acids is both dose- and compound-dependent [9,10,40,41,42]. A proportion is absorbed or hydrolyzed in the upper gastrointestinal tract, whereas the remaining fraction reaches the colon and undergoes extensive microbial transformation [9,10,14,15,40,41,42,44,45,104]. Controlled human studies after coffee exposure have shown the relatively early plasma appearance of intact caffeoylquinic acids and some host-derived metabolites, followed by the later appearance of dihydrocaffeic acid, dihydroferulic acid, and their conjugates, consistent with microbial transformation and subsequent host conjugation [9,40,41]. These microbial metabolites are observed with *t_max* values typically in the range of 4–8 h and with measured *C_max* values ranging from approximately 41 to 385 nmol/L for dihydroferulic and dihydrocaffeic acid in plasma, consistent with substantial colonic microbial generation and subsequent absorption [9,40,41]. In longer-term intervention studies, repeated coffee consumption produces measurable changes in circulating and urinary coffee-related metabolites, although many phenolic-acid derivatives remain present at relatively low systemic concentrations [41,71,108]. Additionally, coffee polysaccharides and melanoidins introduce additional uncertainty because their colonic availability and fermentability depend on the molecular structure, roasting-related modification, phenolic binding, and beverage–matrix interactions [5,6,10,13,44,105].

These pharmacokinetic and structural differences limit direct extrapolation from experimental models to habitual coffee consumption. Cell studies frequently use unconjugated parent compounds at concentrations that may exceed those achieved in human tissues, whereas animal studies often administer purified caffeine, chlorogenic acids, caffeic acid, or coffee extracts at comparatively high doses. Human-equivalent dose conversion does not fully account for interspecies differences in absorption, microbial metabolism, conjugation, tissue distribution, and exposure duration. Such findings should therefore be interpreted primarily as evidence of biological plausibility unless physiologically relevant exposure has been demonstrated [7,18,37,50,53,64,85]. These data should be integrated with plasma or urinary biomarkers, including caffeine and its metabolites—paraxanthine, theobromine, and theophylline—as well as phenolic acid conjugates and other relevant microbial or host–microbial metabolites [9,34,40,41,71,108]. Future interventions should report the coffee type, roasting and brewing conditions, filtration method, serving volume, intake frequency, intervention duration, additives, and measured concentrations of major constituents.

### 9.2. A Biomarker Framework for Exposure, Microbial Response, Host–Microbiome Metabolism, and Clinical Outcomes

A biomarker framework for precision coffee nutrition should distinguish four biologically different domains: coffee-exposure biomarkers, microbial-response biomarkers, intermediate host-microbiome metabolic biomarkers, and clinical-outcome biomarkers. Exposure biomarkers address whether coffee or a coffee-derived constituent was consumed, absorbed, and metabolically available; microbial-response biomarkers address whether the gut microbial community, taxonomic composition, strain characteristics, or functional activity changed; intermediate host-microbiome metabolic biomarkers capture biochemical processes that may connect microbial activity with host physiology; and clinical-outcome biomarkers assess whether these changes are associated with a disease-relevant or patient-important outcome.

An assessment of coffee exposure should document the coffee species or cultivar, roast degree, brewing method, serving size, timing of consumption, filtration method, consumption frequency, use of additives, and estimated intake of caffeine and major phenolic constituents. Established coffee-related analytes, including caffeine, paraxanthine, theobromine, theophylline, chlorogenic acids, and phenolic-acid derivatives, can be quantified using targeted analytical approaches with high specificity [2,3,9,12,34,40,41,71]. In addition, current studies have applied untargeted or semi-targeted high-resolution mass-spectrometry-based metabolomics to identify broader coffee-associated metabolite signatures, which may improve exposure classification when beverage composition is heterogeneous or incompletely documented [71,108]. Different biospecimen types may represent distinct exposure windows: concentrations measured in plasma or serum primarily reflect recent absorption and systemic availability, whereas urinary metabolites reflect recent excretion and, depending on the collection protocol, integrated exposure during the preceding sampling interval. Fecal measurements may provide complementary information on coffee-derived compounds that escape absorption in the upper gastrointestinal tract and become available for microbial transformation in the colon [10,13,14,15,44]. Combining intake records with targeted quantification and broader metabolomic profiling may therefore provide a more reliable estimate of coffee exposure and dose.

Microbial-response markers include changes in microbial taxa, strain-level features, microbial genes, pathways, and metabolites that are associated with coffee exposure. For example, changes in L. asaccharolyticus, microbial fermentation capacity, or the abundance of SCFA- and phenolic-acid-producing pathways may indicate an ecological or functional microbial response [14,15,29,35,36,38,39,44,66,67], but do not by themselves demonstrate that the altered microorganism or pathway mediates a clinical benefit. Intermediate host-microbiome metabolic markers include circulating or fecal SCFAs, microbial phenolic-acid derivatives and their host conjugates, primary and secondary bile acids, intestinal permeability-related measures, LPS or other microbial products, and inflammatory or metabolic signaling markers. These measures may provide a biologically plausible link between microbial activity and host physiology, but their direction, compartment, timing, and association with coffee-related outcomes should be considered carefully. For instance, an increase in a circulating phenolic-acid conjugate may indicate absorption and host metabolism rather than a causal protective effect [9,40,41], while an altered bile acid profile or inflammatory marker may reflect downstream physiology or a complex host–microbiome interaction [16,17,46,47,49,55,72,73,74,75]. A candidate biomarker should be considered a mechanistic mediator only when evidence indicates that coffee exposure precedes changes in the biomarker, the biomarker is associated with the subsequent clinical outcome, and experimental modification of the biomarker or its underlying pathway attenuates, abolishes, or reproduces the coffee-related effect. Longitudinal sampling, randomized or controlled feeding studies, mediation analyses, microbiota-directed interventions, and targeted metabolite supplementation studies may help distinguish causal mediation from correlation. In the absence of such evidence, these measures should be described as candidate response markers or intermediate biomarkers rather than established mechanistic mediators.

Clinical-outcome biomarkers should reflect clinically meaningful phenotypes and should be selected according to the disease context and the intended use of the biomarker panel. In obesity research, relevant outcomes may include the body weight trajectory, energy intake, resting energy expenditure, body composition, visceral adiposity, and longitudinal changes in waist circumference. In MASLD, clinically relevant outcomes may include liver enzymes, liver fat, non-invasive fibrosis scores, transient elastography or liver stiffness, and, where clinically justified, histological measures of steatohepatitis or fibrosis [20,21,93,95,96]. These outcomes should be distinguished from intermediate biomarkers because they represent the final disease-relevant phenotype rather than a presumed biological pathway. The choice of endpoint should also consider the time scale of the proposed mechanism: changes in plasma metabolites or postprandial glycaemia may occur over hours to days, whereas the body composition, hepatic steatosis, fibrosis, and incident T2D generally require longer follow-up. Repeated outcome assessment, standardized phenotyping, and adjustment for baseline disease severity, medication use, habitual diet, physical activity, smoking, alcohol intake, sleep, and coffee preparation are therefore essential for reducing misclassification and residual confounding.

### 9.3. Risk–Benefit Considerations and Safety

Caffeine-related adverse effects can include sleep disruption, anxiety, palpitations, and transient changes in blood pressure or heart rhythm, particularly at higher doses or when coffee is consumed close to bedtime [32,101,109,110]. In a randomized ambulatory trial, caffeinated coffee intake increased daily step counts and premature ventricular contractions but reduced the sleep duration, illustrating that the same exposure may produce favorable and unfavorable short-term effects across different physiological domains [109]. Pregnancy is an important context for risk assessment. Prospective human studies have associated higher maternal caffeine exposure with fetal growth restriction or smaller neonatal anthropometric measurements, although residual confounding cannot be completely excluded [111,112,113]. The preparation method also introduces risks that are not captured by the caffeine content alone. Unfiltered coffee can contain substantially higher concentrations of cafestol and kahweol than paper-filtered coffee, and controlled intervention studies indicate that the sustained consumption of unfiltered coffee can increase total and LDL cholesterol [4,60].

Additives can further alter the overall metabolic effect of coffee beverages. Added sugar and energy-dense creamers increase total energy exposure and may confound associations attributed to coffee itself [82,105]. Non-nutritive sweeteners, emulsifiers, and stabilizers should also be treated as independent exposures: controlled human studies have shown that selected non-nutritive sweeteners can induce person-specific glycemic responses accompanied by microbiome changes, while carboxymethylcellulose can alter microbial composition and metabolomic profiles in some individuals [106,107,114].

## 10. Conclusions and Future Research Directions

Coffee should be considered a heterogeneous dietary exposure rather than a uniform source of caffeine or polyphenols. Caffeine is absorbed predominantly in the upper gastrointestinal tract and undergoes host-mediated metabolism, whereas fractions of chlorogenic acids, non-digestible polysaccharides, and roasting-derived melanoidins reach the colon and undergo microbial fermentation and biotransformation. The resulting SCFAs and phenolic acid metabolites and potentially modified bile-acid profiles may influence epithelial barrier function, mucosal immunity, enteroendocrine signaling, and gut–liver communication. These pathways provide a biologically plausible framework linking coffee exposure with energy balance, glucose regulation, and hepatic lipid and inflammatory pathways. However, the magnitude and clinical relevance of these effects are likely to depend on the coffee composition, dose, preparation method, host metabolism, baseline microbiota, background diet, and metabolic phenotype.

Several important limitations currently prevent firm mechanistic or clinical conclusions. First, many studies have used purified caffeine, chlorogenic acids, extracts, spent coffee grounds, or experimental doses that do not reproduce the chemical composition and exposure levels of routinely consumed brewed coffee. Second, evidence for microbiome responses is often based on relative taxonomic abundance, without parallel measurements of microbial pathways, absolute metabolite production, or metabolic flux. Third, most mechanistic evidence is derived from in vitro systems or animal models, whereas human trials are generally small, short-term, and heterogeneous in the coffee preparation, dose, comparator, dietary background, and clinical phenotype. Finally, associations among coffee intake, microbial features, and cardiometabolic outcomes do not establish that the gut microbiota mediates the observed effects.

Future research should first establish a standardized exposure platform. Multicenter randomized factorial feeding trials should compare chemically characterized caffeinated and decaffeinated coffee with a matched non-coffee control, while controlling the dose, roast degree, brewing and filtration methods, and beverage additives. Acute and longitudinal sampling should integrate coffee-exposure biomarkers, metagenomic or metatranscriptomic profiles, absolute microbial abundance, SCFAs, phenolic-acid metabolites, bile acids, intestinal-barrier markers, GLP-1/PYY, continuous glucose monitoring, insulin sensitivity, resting energy expenditure, body composition, and MRI-derived liver fat. Participants should be stratified according to the baseline microbiota, caffeine-metabolism genotype, metabolic phenotype, and dietary context to identify determinants of inter-individual responses. Causal mediation should then be tested using human-to-mouse microbiota transfer, defined microbial communities, targeted metabolite supplementation, and pathway-specific perturbation or receptor inhibition, followed by adequately powered long-term randomized trials in obesity, T2D, and MASLD with clinically relevant efficacy and safety endpoints. This integrated strategy would determine whether coffee-associated microbial changes are causal mediators or merely biomarkers of exposure and the host phenotype.

## Data Availability

No new data were created or analyzed in this study. Data sharing is not applicable to this article.

## Supplementary Materials

The following supporting information can be downloaded at: https://www.mdpi.com/article/10.3390/metabo16090680/s1.
