# Supplementary material for: Coffee, the Gut Microbiome, and Host Metabolism: Gastrointestinal Fate, Microbial Transformation, Mechanistic Insights, and Prospects for Precision Nutrition
Source: Metabolites. 2026 Sep 16;16(9):680. doi: 10.3390/metabo16090680 (PMC13609105; doi:10.3390/metabo16090680)
Supplement: Supplementary file 1 [file metabolites-16-00680-s001.zip › metabolites-4537272-supplementary.pdf]

Detailed Literature Search and Evidence Synthesis

A non-systematic literature search was conducted in PubMed/MEDLINE and Google Scholar from database inception to 1 July 2026. The core PubMed/MEDLINE search strategy combined terms related to coffee and its major constituents with terms related to the gut microbiota, microbial metabolites, intestinal barrier function, the gut–liver axis, and metabolic disease. Additional targeted searches were conducted by combining coffee-related terms with processing and brewing terms, including roasting, roast profile, Maillard reaction, melanoidin, dietary fiber, pyrolysis, brewing, and coffee species. Additional searches addressed pharmacokinetic and exposure-related terms, including absorption, plasma, urinary, metabolite, exposure, and pharmacokinetics. Disease-specific searches combined coffee-related terms with obesity, adiposity, type 2 diabetes, insulin resistance, MASLD, NAFLD, hepatic steatosis, liver fat, liver stiffness, inflammation, and related metabolic outcomes. The following Boolean search string represents the core PubMed/MEDLINE search strategy:

```
(("Coffee"[Mesh] OR coffee[tiab] OR caffeine[tiab] OR
"chlorogenic acid"[tiab] OR "chlorogenic acids"[tiab] OR
"caffeic acid"[tiab] OR trigonelline[tiab] OR melanoidin*[tiab])
AND
("Gastrointestinal Microbiome"[Mesh] OR microbiota[tiab] OR
microbiome[tiab] OR "gut bacteria"[tiab] OR
"microbial metabolite*"[tiab] OR "short-chain fatty acid*"[tiab] OR
"phenolic acid*"[tiab] OR "bile acid*"[tiab] OR
"gut-liver axis"[tiab] OR "intestinal barrier"[tiab])
AND
(metabolic[tiab] OR obes*[tiab] OR "type 2 diabetes"[tiab] OR
T2D[tiab] OR MASLD[tiab] OR NAFLD[tiab] OR liver[tiab]))
```

Coffee AND (roasting OR roast profile OR Maillard reaction OR melanoidin* OR pyrolysis OR brewing)

Coffee AND (pharmacokinetic* OR absorption OR plasma OR urinary OR metabolite* OR exposure)

Coffee AND (obesity OR adiposity OR "type 2 diabetes" OR insulin resistance)

Coffee AND (MASLD OR NAFLD OR hepatic steatosis OR liver fat OR liver stiffness)

Coffee AND (bile acid* OR short-chain fatty acid* OR phenolic acid* OR gut-liver axis)

Coffee AND (intestinal barrier OR tight junction* OR gut permeability OR endotoxin)

Studies were considered eligible when they addressed at least one of the following:

(i) the composition, digestion, absorption, or metabolism of coffee constituents; (ii) the effects of coffee or defined coffee constituents on the gut microbiota or microbial metabolites; (iii) intestinal barrier function or gut–liver signaling; (iv) associations between coffee exposure and metabolic, inflammatory, or hepatic outcomes; (v) pharmacokinetic or exposure data relevant to human coffee consumption; or (vi) processing- or brewing-related changes that could plausibly modify the gastrointestinal fate of coffee constituents.

Human intervention studies, prospective cohort studies, and pharmacokinetic investigations were prioritized when making statements about exposure, clinical relevance, or translational potential. Animal and in vitro studies were used primarily to describe biological mechanisms and assess mechanistic plausibility; they were not treated as direct evidence of a clinical benefit. Studies were excluded when they did not examine coffee or a clearly defined coffee-derived preparation, focused exclusively on unrelated beverages or food matrices, did not address biological outcomes relevant to this review, or provided insufficient information to support the specific claim under consideration. Studies involving isolated caffeine, purified chlorogenic acids, coffee extracts, spent coffee grounds, coffee pulp, or other coffee-derived preparations were retained when mechanistically informative, but their findings were explicitly distinguished from evidence obtained with whole brewed coffee. Earlier studies using the term NAFLD were retained when relevant and interpreted within the current MASLD framework. Additionally, For the disease-specific sections, evidence was interpreted according to a predefined hierarchy. Observational studies were used to describe population-level associations but not to infer causality, randomized clinical trials were given priority for estimating intervention effects, animal studies were used to assess organism-level biological effects and pathway plausibility, and cell or in vitro studies were used to examine molecular or microbial mechanisms under controlled conditions. Mechanistic interpretations were considered hypothesis-generating unless supported by temporally ordered human data, mediation analyses, or microbiota-directed experimental evidence. Null or unfavorable findings from randomized trials were retained and discussed alongside positive observational and preclinical findings rather than being treated as secondary evidence.

Records were screened for relevance at the title and abstract level, followed by a full-text assessment when the relevance, study design, or evidence level could not be determined from the abstract. Reference lists of relevant primary studies and review articles were screened manually, and citation tracking was used to identify additional studies. Studies were prioritized according to their direct relevance to the review question, methodological contribution, human translational value, recency where

appropriate, and mechanistic informativeness. For overlapping or repeated evidence, the most complete, methodologically informative, or directly relevant report was prioritized. When multiple publications arose from the same cohort or experimental dataset, they were considered together to avoid presenting the same evidence as independent replication. L.Y. and C.W conducted the initial relevance assessment and subsequently review.
